# Oxidative Stress in the Developing Rat Brain due to Production of Reactive Oxygen and Nitrogen Species

**DOI:** 10.1155/2016/5057610

**Published:** 2016-04-13

**Authors:** Jiří Wilhelm, Richard Vytášek, Jiří Uhlík, Luděk Vajner

**Affiliations:** ^1^Institute of Physiology, Czech Academy of Sciences, Vídeňská 1083, Krč, 14220 Prague 4, Czech Republic; ^2^Department of Histology and Embryology, 2nd Faculty of Medicine, Charles University in Prague, Plzeňská 311/221, Motol, 15000 Prague 5, Czech Republic

## Abstract

Oxidative stress after birth led us to localize reactive oxygen and nitrogen species (RONS) production in the developing rat brain. Brains were assessed a day prenatally and on postnatal days 1, 2, 4, 8, 14, 30, and 60. Oxidation of dihydroethidium detected superoxide; 6-carboxy-2′,7′-dichlorodihydrofluorescein diacetate revealed hydrogen peroxide; immunohistochemical proof of nitrotyrosine and carboxyethyllysine detected peroxynitrite formation and lipid peroxidation, respectively. Blue autofluorescence detected protein oxidation. The foetuses showed moderate RONS production, which changed cyclically during further development. The periods and sites of peak production of individual RONS differed, suggesting independent generation. On day 1, neuronal/glial RONS production decreased indicating that increased oxygen concentration after birth did not cause oxidative stress. Dramatic changes in the amount and the sites of RONS production occurred on day 4. Nitrotyrosine detection reached its maximum. Day 14 represented other vast alterations in RONS generation. Superoxide production in arachnoidal membrane reached its peak. From this day on, the internal elastic laminae of blood vessels revealed the blue autofluorescence. The adult animals produced moderate levels of superoxide; all other markers reached their minimum. There was a strong correlation between detection of nitrotyrosine and carboxyethyllysine probably caused by lipid peroxidation initiated with RONS.

## 1. Introduction

Reactive oxygen species (ROS) such as hydrogen peroxide, superoxide, and hydroxyl radicals together with reactive nitrogen species (RNS) derived from nitric oxide constitute common intermediates of cellular metabolism and they are collectively referred to as RONS. Their overproduction is the cause of the oxidative stress; however, at very low concentrations, they can function as signaling molecules [1, 2]. Oxidative stress participates in many pathologies and is also considered a major factor in the ageing process [3]. Brain reveals high specific oxygen consumption due to abundant mitochondria and highly active respiratory enzymes [4]; therefore, the effect of oxidative stress on brain ageing is widely recognized [5]. Nitric oxide (NO) is enzymatically synthesized by three isoforms of nitric oxide synthase, that is, neuronal (nNOS), endothelial (eNOS), and inducible (iNOS). It was shown that transcripts for all isoforms are present in the brain during embryonal and postnatal development [6].

Brain oxygen concentration highly increases after birth compared to the foetus, thus creating a condition of oxidative stress. The expression of both nNOS and iNOS increases after birth reaching a peak on the fifth postnatal day [7]. Excessive NO production injures the adjacent tissues by formation of peroxynitrite which originates from the rapid interaction with superoxide. These reactions result in the formation of nitrotyrosine moieties in affected proteins. Nitrotyrosine was detected in the cortex of aging rats [8] and also during the early postnatal period [7]. Besides that, the early postnatal period is characterized by apoptotic elimination of both neurons and glia which proliferated in overabundance [9]. Such an elimination and further proliferation of glial and even nerve cells manifest some phasing during the first two postnatal months [10]. Apoptotic cells are phagocyted by microglia with concomitant production of RONS. Superoxide was detected by staining the living brain slices with nitroblue tetrazolium, and microglial respiratory burst was detected in vivo using a fluorescent probe [11]. Peroxynitrite can also initiate membrane lipid peroxidation [12, 13] and catalyze glycoxidation and lipoxidation reactions [14]. Thus, the products of glycoxidation reactions such as carboxyethyllysine may be formed in the sites of NO-induced lipid peroxidation [15].

The increased production of free radicals in the early postnatal period suggests that oxidative stress is generally occurring after normal birth. An important study in this regard showed ROS-mediated oxidative damage to DNA in rat liver, kidney, and skin during the first few hours after normal birth. The lesions were considered substantial, having been similar to or exceeding the levels in 24-month-old rats [16]. This concept was further supported by the finding of pronounced neonatal decreases in the hepatic glutathione/glutathione disulfide (GSH/GSSG) ratio in rats [17, 18]. Also, the product of membrane lipid peroxidation, malondialdehyde, exhibited a transient rise after birth in rat liver and kidney [19]. In the guinea pig brain, the increase in lipid peroxidation was observed during prenatal period [20]. Studies in the rat brain recognized proteins modified by lipid peroxide decomposition product, 4-hydroxynonenal (HNE), by immunocytochemistry and electron microscopy. A moderate increase in labeling was observed in the corpus callosum in the 7-day-old and 8-day-old rats, on day 10 it was maximum, sharp decrease was observed on day 14, and no staining was observed in adult rats. Electron microscopy showed dense HNE staining on the basal laminae of blood vessels and on the plasma membranes of unmyelinated axons [21]. In the human brain, HNE protein adducts increased in parietal white matter from gestational week 40 to postnatal year 1.5 encompassing the peak period of myelin sheath synthesis at this site. It was concluded that human brain development involves basal levels of oxidative stress [22]. In the recent study [23], ROS production was found to increase in developing mouse heart from postnatal day 1 to day 7, when it was maximal, and correlated with DNA oxidative damage.

In a preceding work we analyzed the quantitative aspects of free radicals production in the developing rat brain [24]. We have documented the usefulness of redox-sensitive fluorescent probes in connection with fluorescence microscopy for the detection of RONS in vivo in further studies [25, 26]. In the present study, we have focused our aim at the localization of the sites of RONS production. We used dihydroethidium (DHE) for the detection of superoxide and 6-carboxy-2′,7′-dichlorodihydrofluorescein diacetate (H2DCFDA) as a probe for hydrogen peroxide. Antibodies against protein nitrotyrosine detected the results of nitric oxide overproduction, antibodies against carboxyethyllysine localized the sites of nonenzymatic glycation and lipid peroxidation, and blue autofluorescence detected oxidized proteins. This integrative approach has enabled the detailed localization of the sites of RONS production in the developing brain.

## 2. Materials and Methods

### 2.1. Animals

The study was conducted in accordance with the Guide for the Care and Use of Laboratory Animals published by the US National Institutes of Health (NIH publication number 85-23, revised 1996). Experiments were approved by the Animal Protection Expert Commission of the Faculty.

Twelve pregnant female Wistar rats were used in the experiments. They had free access to water and standard laboratory diet. The offspring (a total of 65 neonates) of both sexes were investigated 1 day before birth (group F, *n* = 12, mean weight: 4.32 ± 0.30 g) and then on days 1 (group D1, *n* = 10, mean weight: 6.29 ± 0.41 g), 2 (group D2, *n* = 10, mean weight: 7.91 ± 0.23 g), 4 (group D4, *n* = 10, mean weight: 10.15 ± 0.99 g), 8 (group D8, *n* = 8, mean weight: 16.64 ± 2.00 g), 14 (group D14, *n* = 7, mean weight: 25.68 ± 1.79 g), 30 (group D30, *n* = 4, mean weight: 110.00 ± 2.5 g), and 60 (group D60, *n* = 4, mean weight: 275.15 ± 7.07 g) of postnatal life. The preliminary studies did not reveal any gender difference in structures of interest; therefore, distribution of males and females in groups was random.

On the sampling day, rats were weighed and sacrificed under general anaesthesia (Thiopental, 40 mg kg^−1^ b.w., i.p., VUAB Pharma a.s., Roztoky, Czech Republic) by decapitation (groups F, D1, D2, D4, and D8) or by cutting the spinal cord (groups D14, D30, and D60). In F group, mothers were anaesthetized as well. Isolated brains were cut transversally approximately at the bregma level, snap-frozen in the liquid nitrogen, and stored at −80°C.

### 2.2. Detection of ROS Production by Fluorescence Microscopy

Brains were cut at −20°C using the cryostat Leica CM 1950 (Leica Microsystems, Germany) at 5 *μ*m. The nonfixed sections were used for the experiments. Slides were assessed by the Olympus BX 53 microscope in the bright field and by epifluorescence (filter U-FUW: excitation 340–390 nm, barrier 420 nm; filter U-FBW: excitation 460–495 nm, barrier 510 nm; filter B/G: excitation 481–520 nm, barrier 568–643 nm), respectively. Microphotographs were taken by the ProgRes C5 digital camera using the NIS Elements AR software (Laboratory Imaging, Czech Republic).

For the detection of hydrogen peroxide, we used 6-carboxy-2′,7′-dichlorodihydrofluorescein diacetate (H2DCFDA; Molecular Probes, USA) as specified in our previous work [24]. In brief, the stock solution of H2DCFDA was made by dissolving 5 mg of the dye in 1 mL of ethanol; for staining, it was diluted to 10 *μ*g/mL in phosphate-buffered saline, pH 7.4 (PBS). Slides with frozen brain sections were washed in PBS and incubated with H2DCFDA for 10 min.

A similar procedure was used for the detection of superoxide using dihydroethidium (DHE; Molecular Probes, USA). The stock solution of DHE was made by dissolving 5 mg of the dye in 1 mL of water; for staining, it was diluted to 5 *μ*g/mL in PBS.

All sections (except for autofluorescence) were counterstained with DAPI (4′,6-diamidino-2-phenylindole dihydrochloride; 0.5 *μ*g/mL, 2 min, Sigma-Aldrich Chemie, Germany).

For autofluorescence measurements the thin sections were mounted after PBS washing only.

Finally, sections were mounted into Fluoroshield mounting medium (Sigma-Aldrich Chemie, Germany) under a coverslip and sealed with a lacquer.

Microphotographs were taken by a camera in the bright field under UV to reveal autofluorescence and nuclei stained by DAPI and under appropriate filter to reveal oxidized H2DCFDA or DHE fluorescence. The figures showing fluorescent nuclei were electronically merged with figures showing the specific fluorescence. To elucidate specific structures, some figures taken in the bright field were merged with the figures showing the respective fluorescence.

### 2.3. Immunohistochemistry of Nitrotyrosine and Carboxyethyllysine

Formalin-fixed and paraffin-embedded brain sections cut at 4 *μ*m were used for immunohistochemistry. Three deparaffined and rehydrated tissue sections from each group were treated with 3% H_2_O_2_ in methanol for 30 minutes to block endogenous peroxidases. The washed sections were incubated with the primary mouse monoclonal antibodies NO-60-E3 (against protein-bound 3-nitrotyrosine) diluted 1 : 100 [27, 28] or CEL-9-H11 (against N^*ε*^-(carboxyethyl)lysine) diluted 1 : 200 for 90 min [29]. PBS with 10 mg/mL bovine serum albumin was used for dilution of all antibodies as well as for washings between the incubation steps. Washed sections were incubated with the secondary antibody against mouse IgG labeled with horseradish peroxidase (P260, DakoCytomation, Denmark) diluted 1 : 100. The bond was visualized using diaminobenzidine (Sigma FAST*™* DAB Peroxidase Substrate Tablets, Sigma-Aldrich Chemie, Germany); the reaction was enhanced by 1% CuSO_4_. The sections were counterstained with hematoxylin and mounted into the Solacryl BMX permanent mounting medium. Negative controls were provided by omission of the primary antibody.

## 3. Results

### 3.1. Detection of Superoxide Production with DHE

Oxidation products of DHE were used for the detection of superoxide generation giving orange fluorescing fluorophores. These products can also bind to DNA and as we have counterstained nuclei with the blue fluorescing DAPI, we can encounter in the nuclei three situations: (1) the fluorescence intensity of DHE oxidation products is higher than fluorescence of DAPI and the nucleus will appear as red; (2) the fluorescence intensities of both DHE oxidation products and DAPI are about the same and the resulting light composed of orange and blue will be seen as white; (3) no oxidation of DHE in the vicinity of a nucleus results in the blue fluorescence.

**Figure 1:**
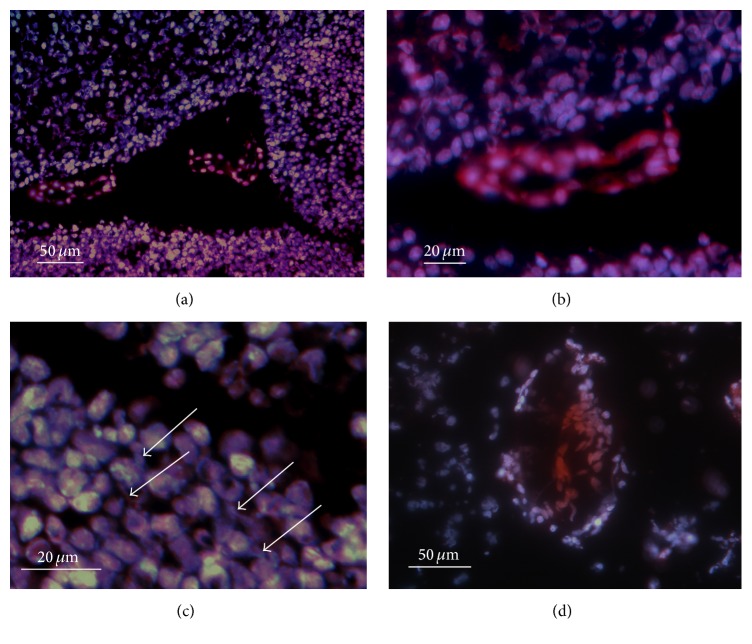
DHE oxidation in the perinatal brain (colocalization with DAPI). (a) Overview of a lateral ventricle between fimbria of the hippocampus and amygdala of the foetal brain. (b) A detailed view of a blood vessel. (c) A detail of the active region; arrows indicate the positive dendrites. (d) The situation on D1.

This situation is illustrated in Figure 1 for the foetal brain (panels (a)–(c)). Panel (a) shows lateral ventricle between fimbria of the hippocampus and amygdala. There are whole regions containing blue, red, and white fluorescing nuclei of neurons and glia, respectively. As it is apparent from the detailed view of a blood vessel in panel (b), the cytoplasm of the endothelial and smooth muscle cells is positive as well, not only the respective nuclei. Panel (c) shows the positive region of panel (a) in greater detail. Indicated by arrows are the neuronal dendrites containing high amount of DHE oxidation products. The situation has changed quickly on D1 (panel (d)). The positivity of cytoplasm of neurons and glial cells was reduced; still positive was the cytoplasm of endothelial cells.

**Figure 2:**
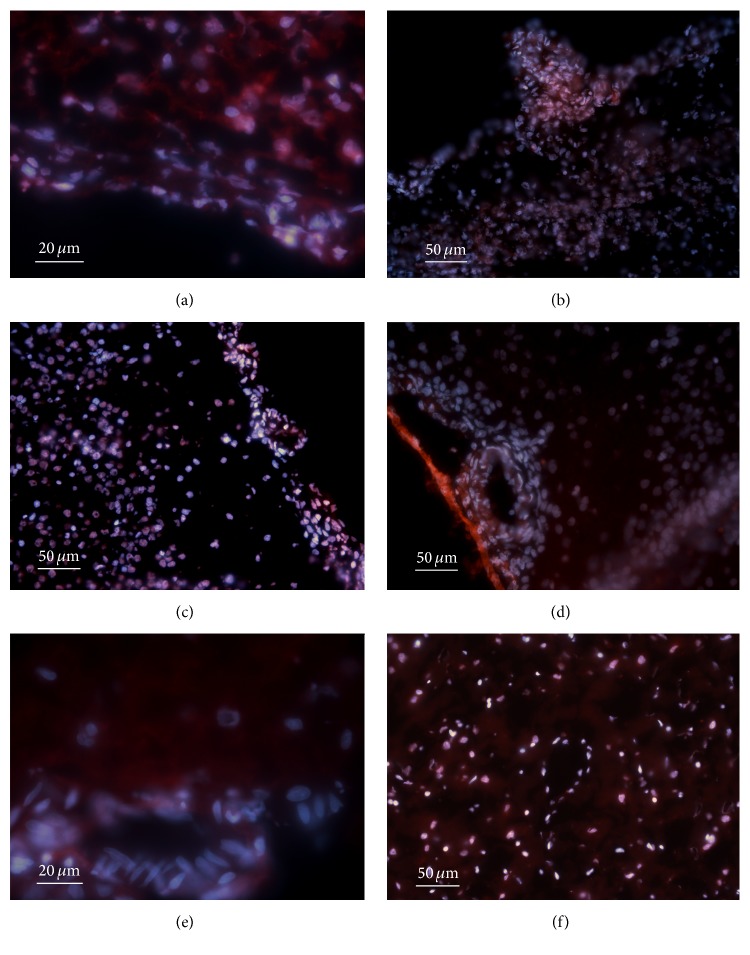
DHE oxidation during brain development (colocalization with DAPI). (a) D2, (b) D4, (c) D8, (d) D14, (e) D30, and (f) D60.

On D2 (Figure 2(a)), the positivity of blood vessels changed in comparison with the foetal brain (compared to Figure 1(b)). The cytoplasm of the endothelial cells was less positive and fluorescence intensity of the neuronal cytoplasm and glial cytoplasm was increased. Panel (b) illustrates both high positivity of the endothelial cells in a blood vessel localized within pia mater and cortical cellular positivity limited to the external granular layer on D4. Panel (c) shows increased positivity observed in the nuclei localized both in the cortex and in the endothelial cells on D8. The red fluorescence was also apparent in the cytoplasm of the respective cells. D14 (panel (d)) was characterized by a total lack of fluorescence in the blood vessel cell cytoplasm and nuclei. The cytoplasm of neurons and glia showed some red fluorescence; however, the highest intensity of bright orange fluorescence was found in the arachnoidal membrane cells. On D30 (panel (e)), the positivity in the blood vessel cells was partially restored and the fluorescence in the neuronal cytoplasm and glial cytoplasm was increased in comparison with D14. All the cortex nuclei demonstrated white to red fluorescence; the cytoplasm was moderately and uniformly active on D60 (panel (f)).

### 3.2. Detection of Hydrogen Peroxide Production with H2DCFDA

**Figure 3:**
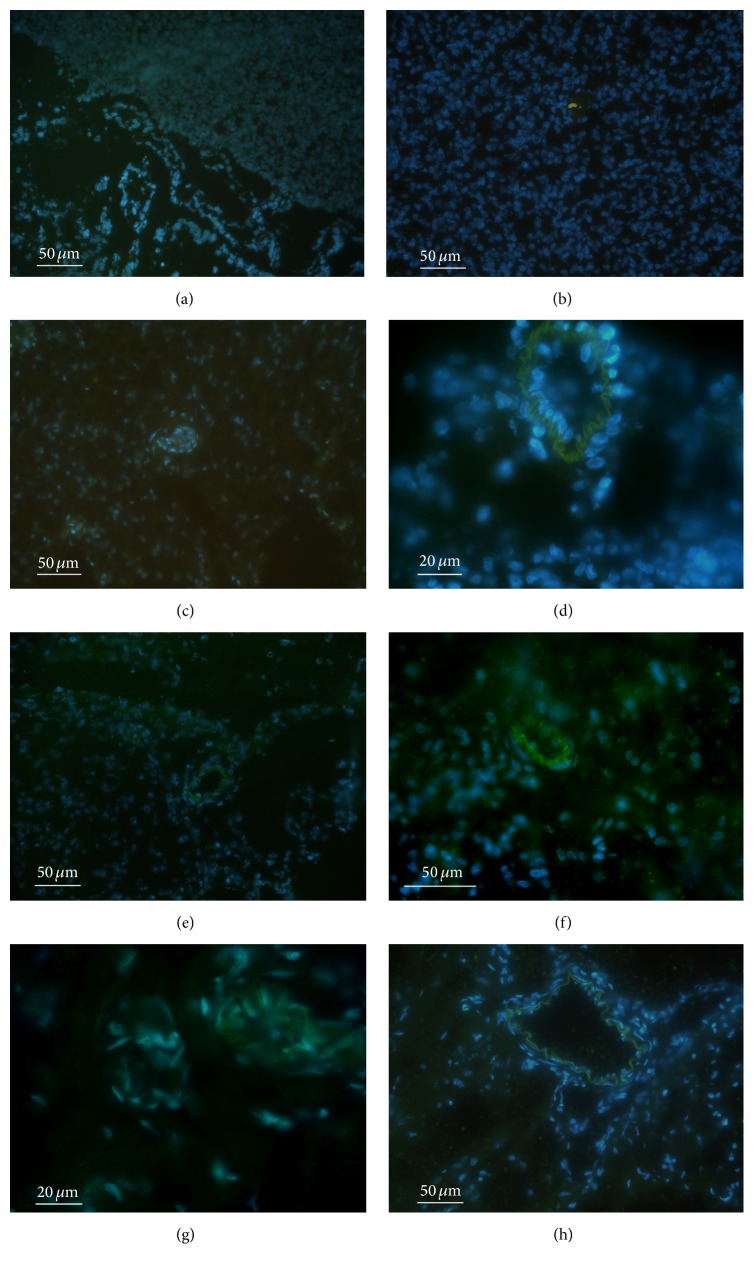
Oxidation of H2DCFDA during brain development (colocalization with DAPI). (a) foetal brain, (b) D1, (c) D2, (d) D4, (e) D8, (f) D14, (g) D30, and (h) D60.

Oxidized H2DCFDA shows green fluorescence which appears as yellow-green at high intensity. The situation in the foetal brain is illustrated in Figure 3(a). We can see uniformly low intensity fluorescence, which is not different in the blood vessel wall cells. On D1, fluorescence in neuronal cytoplasm and glial cytoplasm was without change; however, cells of developing blood vessels appeared intensively bright (panel (b)). A moderate fluorescence could have been observed both in the blood vessel wall cells and in the neuronal cytoplasm and glial cytoplasm on D2 (panel (c)). A distinguished increase in H2DCFDA fluorescence occurred in the blood vessel wall cells on D4 (panel (d)). Intensive fluorescence originated from the internal elastic membrane. Fluorescence intensity in the neuronal cytoplasm and glial cytoplasm was approximately on the same level as on D2. A cessation of fluorescence intensity in the cortical cells was observed on D8; low intensity fluorescence was retained in pia mater and in the blood vessel wall (panel (e)). Fluorescence intensity in the blood vessel wall on D14 was about the same magnitude as on D8; however, new fluorescence appeared in the cytoplasm of neurons and glia (panel (f)). On D30, the intensity of fluorescence of internal elastic membrane was higher than that on D14; fluorescence in the cytoplasm of neurons and glia was practically the same (panel (g)). Faint but still apparent fluorescence can be observed in the brain on D60 (panel (h)).

### 3.3. Localization of the Sites of Protein Nitration with Antibodies against Protein Nitrotyrosine

**Figure 4:**
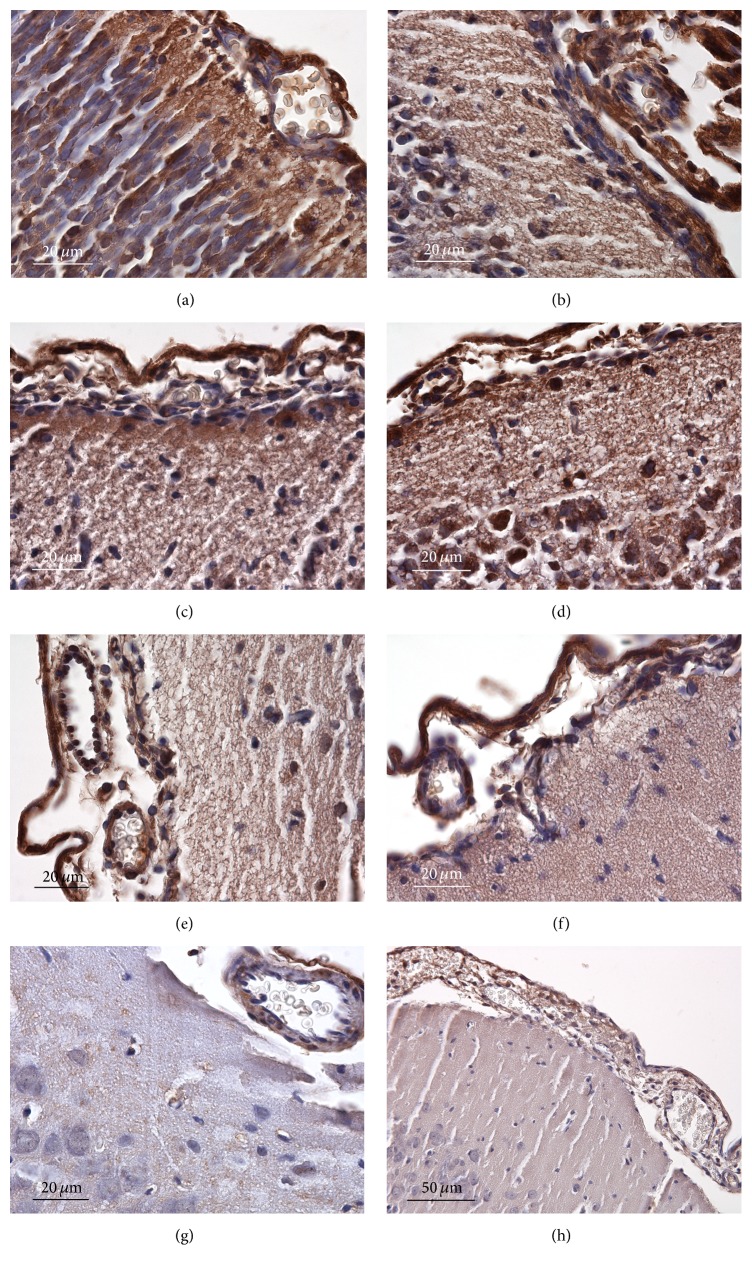
The summary of immunohistochemical detection of nitrated proteins during brain development. (a) foetal brain, (b) D1, (c) D2, (d) D4, (e) D8, (f) D14, (g) D30, and (h) D60.

Production of reactive nitrogen species results in the formation of protein-bound nitrotyrosine (NT) and nitrated proteins can be detected by specific antibodies. The summary of immunohistochemical detection of nitrated proteins in the brain is given in Figure 4. A lot of positive sites can be found in the foetal brain: cytoplasm of most neurons including their dendrites as well as glia, some nuclei, and cytoplasm of endothelial cells (panel (a)). The situation was practically without change on D1 (panel (b)). There was a marked positivity in superficial glial limiting membrane on D2; cytoplasm of neurons, glia, and blood vessel wall cells was without change (panel (c)). On D4, there was the highest extent of positivity found in the whole time range of the study. Cortical neurons and glia in molecular, external granular, and external pyramidal layers were intensively stained. Blood vessel wall was not changed in comparison with D2; however, border arachnoidal cells were highly positive (panel (d)). D8 was characterized by cessation of positivity in neuronal cytoplasm and glial cytoplasm; endothelial cells and border arachnoidal cells maintained high staining intensity (panel (e)). The decrease in the intensity of staining of neurons/glia continued on D14; for the first time, a decrease in the staining intensity in the cytoplasm of endothelial cells was observed. Border arachnoidal cells still stained intensively (panel (f)). The intensity of staining further decreased on D30 and the decrease concerned also the border arachnoidal cells, though their intensity of staining was still highest in comparison with other sites (panel (g)). The situation on D60 was without change in relation to D30 (panel (h)).

### 3.4. Localization of the Sites of Protein Glycation and Lipid Peroxidation with Antibodies against Carboxyethyllysine

**Figure 5:**
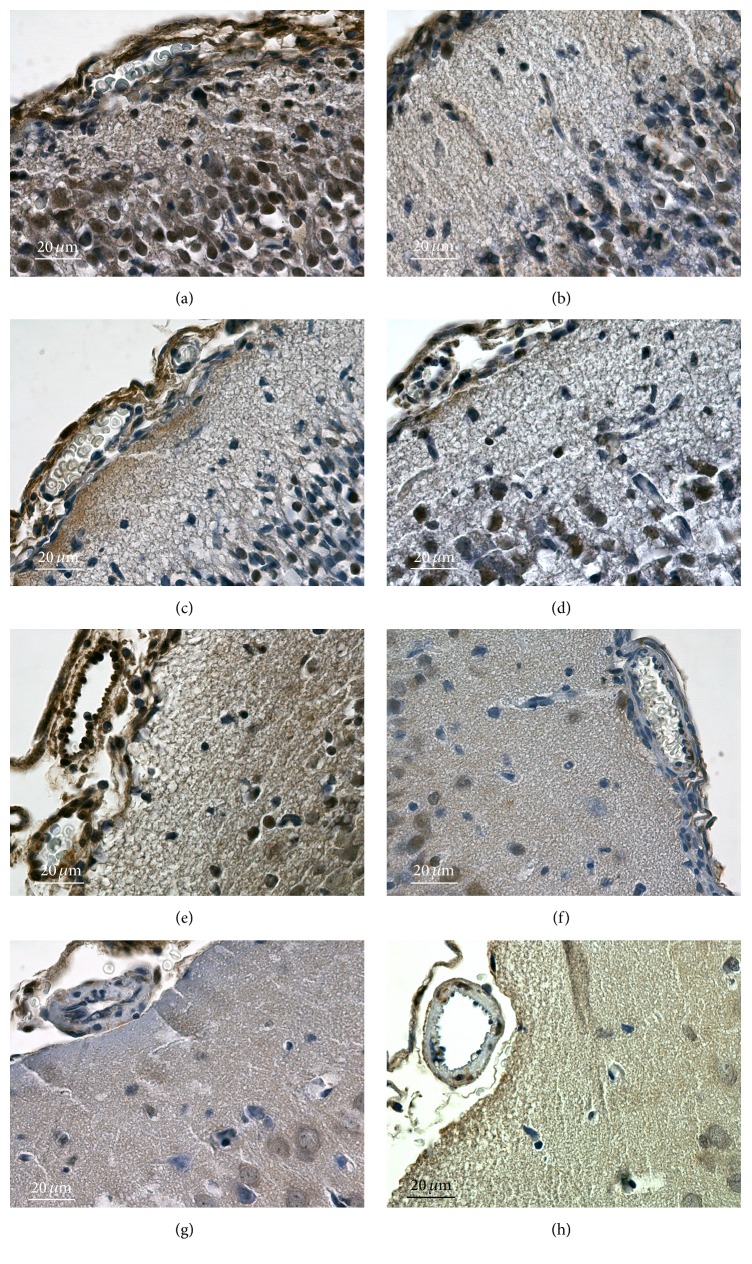
Immunohistochemical detection of carboxyethyllysine during brain development. (a) foetal brain, (b) D1, (c) D2, (d) D4, (e) D8, (f) D14, (g) D30, and (h) D60.

Carboxyethyllysine (CEL) is a product that originates from both protein glycation and lipid peroxidation. As such it represents an important marker of free radical activity. The summary of its immunohistochemical detection in brain is presented in Figure 5. Several positive sites can be found in the foetal brain, especially the cytoplasm of endothelial cells and neurons/glia, the fibrous component of pia mater, and nuclei of neurons/glia in external granular layer (panel (a)). Little less intensive staining was observed on D1 (panel (b)). D2 differs from D1 by increased positivity in border arachnoidal cells; markedly positive was superficial glial limiting membrane (panel (c)). On D4, new cortical positivity appeared in cytoplasm and nuclei of neurons/glia localized in molecular, external granular, and external pyramidal layers. Other sites were without change (panel (d)). More intensive staining of the border arachnoidal cells was the major change on D8 in comparison to D4 (panel (e)). D14 was characterized by a general decrease in the intensity of staining (panel (f)). This decrease continued on D30; positive staining was found in pia mater; border arachnoidal cells and blood vessels showed decreased staining (panel (g)). This pattern continued to D60 (panel (h)).

### 3.5. Localization of Blue Autofluorescence

**Figure 6:**
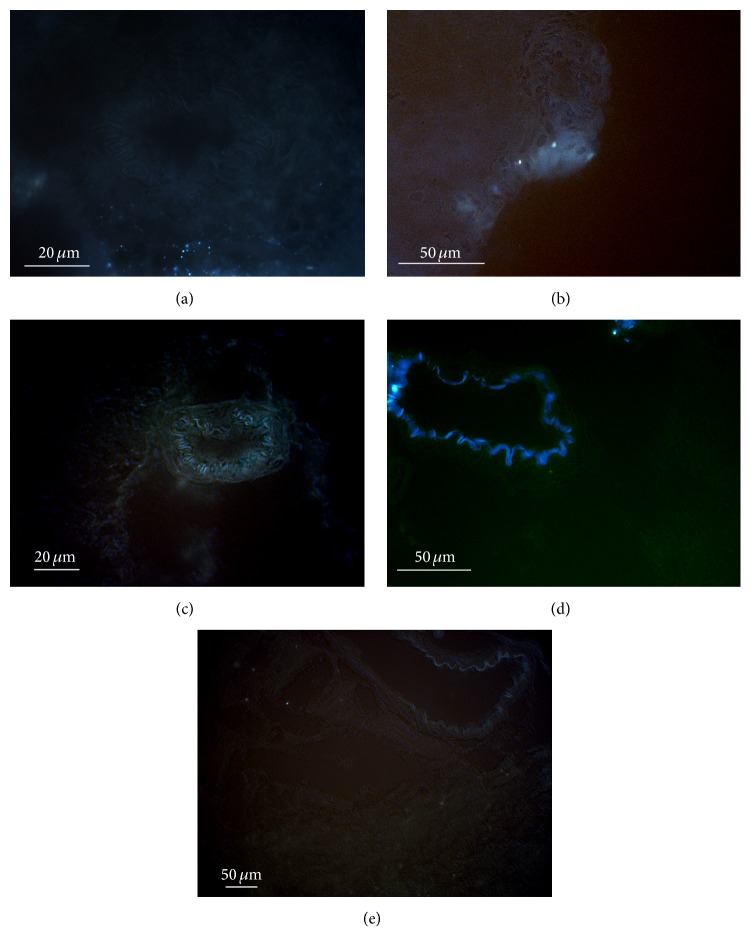
Occurrence of blue autofluorescence during brain development. (a) D4, (b) D8, (c) D14, (d) D30, and (e) D60.

Blue autofluorescence did not appear until D4. Its following time course is illustrated in Figure 6. Isolated areas of pia mater showed low intensity of blue autofluorescence on D4 (panel (a)) and on D8 (panel (b)). No fluorescence of blood vessels was encountered on these days. Starting from D14, the vessel wall was the site of intensive blue fluorescence (panel (c)). The highest intensity of blue fluorescence was observed in the vessel wall on D30 (panel (d)). It was so bright that the sensitivity of the camera had to be reduced which erased the low level fluorescence outside the vessel wall. On D60, the blue autofluorescence in the vessel wall decreased and higher sensitivity of the camera revealed low level autofluorescence in other parts of the brain (panel (e)).

## 4. Discussion

The reaction of dihydroethidium is considered to be relatively specific for superoxide, with minimum reaction with hydrogen peroxide [30]. DHE undergoes a two-electron oxidation to form a DNA-binding fluorophore. In the case of mitochondrial generation of superoxide it binds mitochondrial DNA; at higher concentrations it can bind nuclear DNA [31]. DHE oxidation yields two fluorescent products, 2-hydroxyethidium (EOH), which is more specific for superoxide, and the less specific product, ethidium. Both EOH and ethidium are fluorescent in the absence of DNA; however, in the presence of DNA, their fluorescence is highly increased [32]. We have documented fluorescence due to DHE oxidation by superoxide in the nuclear membrane devoid of DNA which contributed to staining of the nuclei [25].

The oxidation of 2′,7′-dichlorofluorescein (DCFH) to fluorescent dichlorofluorescein (DCF) was originally used for the detection of hydrogen peroxide [33]. For the intracellular detection, use is made of the diacetate form of DCFH (DCFH-DA). DCFH-DA is taken up by the cells and then deacetylated by intracellular esterases and the resulting DCFH becomes trapped inside the cell being ready for the oxidation. However, intracellularly formed DCFH can escape to extracellular space, where it is accessible by extracellular oxidants [34]. Besides hydrogen peroxide, DCFH can be oxidized by peroxidases, even in the absence of hydrogen peroxide [35]. In addition to peroxidase-dependent oxidation of DCFH, other substances can produce DCF in the absence of H_2_O_2_. These substances comprise lipid peroxides [36] and reactive nitrogen species such as peroxynitrite [37]. We have used DCFH for the detection of hydrogen peroxide production in rat heart in our previous studies [25, 26]. In the present study, we employed carboxy-derivative H2DCFDA, which proved to be a more sensitive stain for intracellular hydrogen peroxide production.

Exposure of proteins to free radical-producing systems induces appearance of blue autofluorescence [38, 39]. In a previous study, we have observed blue fluorescence in isolated collagen I exposed to UV irradiation in vitro [40]. Using confocal microscopy, elastin autofluorescence was found in mesenteric arteries of 10-day-old rats. The intensity of fluorescence was much higher in 1-month-old and 6-month-old animals [41].

In the rat hearts, we have observed first signs of autofluorescence of the coronary vessels internal elastic lamina in 7-day-old heart, albeit very faint. On day 15, the autofluorescence was bright enough to be photographed, and the fluorescence intensity reached maximum on day 60. It was still highly pronounced in 7-month-old animals. From day 60 on, there was also observable autofluorescence originating from the cells. To the opposite of the fluorescence of the vessel wall, this fluorescence was extractable to chloroform and its intensity was increasing with age and was higher in 7-month-old animals. We have suggested that this cellular autofluorescence originated from the lipofuscin-like pigments, the end products of lipid peroxidation [26].

As the individual stains reveal specific parts of the general free radical mediated processes, the comparison of their time course can provide an insight into the mechanisms of the oxidative stress during brain development. The foetal brain just before birth contained several markers of active free radical generation. Superoxide production could have been found in many sites, while production of hydrogen peroxide was relatively lower. Production of RONS is revealed by increased staining for NT in the cytoplasm and nuclei of neurons/glia and endothelial cells. It corresponded to CEL staining which could have been the result of lipid peroxidation in the areas of RONS production. According to Bandeira et al. [10], more than 90% of the brain cells are neurons in this period.

Immediately after birth on D1, there was a decrease in neuron/glia superoxide production which was accompanied by low level of hydrogen peroxide. It thus appears that increased oxygen concentration after birth does not contribute to increased ROS generation in neurons/glia. The first three days represent dormant phase concerning cell proliferation [10]. On the other hand, the marked change was observed in the vessel wall which intensively produced hydrogen peroxide, and endothelial cells actively generated superoxide. This could have been related to the intensive metabolism of developing blood vessels. The amount of NT was without change in comparison to foetuses; CEL was decreased a little.

Dynamic changes in RONS production appeared on D2. Production of superoxide in neurons/glia increased, while H_2_O_2_ production stayed low. Endothelial cells produced less superoxide and blood vessels showed lowered production of H_2_O_2_, indicating that metabolic activity in blood vessels is not increasing linearly. The amount of NT in neurons/glia and endothelial cells was practically without change. Positivity in superficial glial limiting membrane appeared newly as well as intense positivity for CEL. So this is the possible site of lipid peroxidation initiated by RONS.

With regard to RONS production, D4 appears to be the critical point in development. Superoxide production by endothelial cells was high again, accompanied by intense production of hydrogen peroxide in the vessel wall. The most pronounced positivity was observed in the vessel internal elastic membrane. In our previous study on rat hearts we found that H_2_O_2_ in the internal elastic membrane is produced by lysyl oxidase [26]. The neuronal/glial superoxide production decreased a little; the most significant change, however, was in the site of production, as cortical positivity was limited to the external granular layer. The striking increase was observed in NT staining which was highest throughout the whole development. From this time point on, the intensity of NT staining was decreasing. Newly appearing CEL positivity in neuronal/glial cytoplasm could have been associated with increased RONS production, again. D4 was also the first time period when blue autofluorescence appeared. This might have been the result of lipid peroxidation. From D4 till D7, number of neurons duplicated and the brain volume increased due to neuronal growth as well [10].

Superoxide production was restarted in cortical neurons/glia on D8, accompanied by decreased production of H_2_O_2_. Increased NT staining was maintained in the cytoplasm of endothelial cells and border arachnoidal cells, which also stained for CEL and showed blue autofluorescence. These two parameters might indicate initiation of lipid peroxidation.

During the second and third weeks, glial cells proliferated fifty times more, while number of neurons decreased by 70% during the second week [10]. D14 represented another period when the character of RONS production has changed dramatically. There was no indication of superoxide production in the blood vessel cell cytoplasm and nuclei. On the other hand, highest level of superoxide, which was maximal throughout the development, was found in the arachnoidal membrane. In contrast to superoxide, H_2_O_2_ production in the cytoplasm of all cells was on the highest level in the development. Moderate H_2_O_2_ production was observed in the internal elastic lamina which for the first time in the development gave blue autofluorescence. Both NT and CEL were generally decreased.

New increase in superoxide production in neurons/glia and blood vessel cells was found on D30, accompanied with increased H_2_O_2_ production in the internal elastic membrane. Also, the blue autofluorescence of the internal elastic membrane was the most intensive in the whole development. NT staining further decreased and the decrease concerned also the border arachnoidal cells; CEL continued to decrease.

From D25 till D60, the second wave of growth came [10]. A moderate superoxide production was found in all cortex cells on D60, the levels of all other markers reached their minimum, and the blue autofluorescence was less intensive than that on D30.

The generation of superoxide and hydrogen peroxide in the developing brain had a cyclical character. The periods of their production have differed as well as the sites of maximum production, indicating that they have been generated by independent processes. DCFH has been shown to be an indicator of peroxynitrite in vitro [42]; however, maximal oxidation of H2DCFDA in our experiments did not correlate with NT staining; thus, it appears that H2DCFDA oxidation is influenced by peroxynitrite only a little in the developing brain. On the other hand, there was a strong correlation between staining for NT and that for CEL, which suggests that CEL could have been produced in lipid peroxidation initiated with RNS. This view is also supported by the appearance of cellular blue autofluorescence. Generation of blue autofluorescence in the internal elastic membrane of the blood vessels started on D14, reached a peak on D30, and decreased on D60. This behaviour differs from that in the heart, where the maximum was found on D60 [26]. It indicates that blood vessels develop in the heart and brain in a specific way, although both processes involve hydrogen peroxide.

A comparison of these events to events of the brain growth [10] shows a time correlation, especially with the end of the postnatal neurogenesis by D7-8 and starting gliogenesis by the second week. During the third week, late neurogenesis takes place. It is important to note that the output of pathologic or toxicologic studies in young animals might depend on the timing, as the periods of intrinsic oxidative stress would modify the response to other treatments.

This study documents that oxidative stress is an inherent component of normal postnatal brain development and it has a characteristic time course, closely related to growth and apoptotic changes. The output of additional external stress factors might be dependent on their timing, that is, whether they appear during a period of high or low free radical production. The oxidative damage to proteins with a long half-life early in the development, such as elastin and collagen which was detected by blue fluorescence, suggests that these effects might be revealed later in life and even influence the animal longevity.

## Abbreviations

RONS: Reactive oxygen and nitrogen species
ROS: Reactive oxygen species
RNS: Reactive nitrogen species
NO: Nitric oxide
nNOS: Neuronal nitric oxide synthase
eNOS: Endothelial nitric oxide synthase
iNOS: Inducible nitric oxide synthase
GSH: Glutathione
GSSG: Glutathione disulfide
HNE: 4-Hydroxynonenal
DHE: Dihydroethidium
H2DCFDA: 6-Carboxy-2′,7′-dichlorodihydrofluorescein diacetate
PBS: Phosphate-buffered saline, pH 7.4
DAPI: 4′,6-Diamidino-2-phenylindole dihydrochloride
NT: Nitrotyrosine
CEL: Carboxyethyllysine
EOH: 2-Hydroxyethidium
DCFH: 2′,7′-Dichlorofluorescein
DCF: Dichlorofluorescein
DCFH-DA: Dichlorofluorescein diacetate.
